# HELLS is required for maintaining proper DNA modification at human satellite repeats

**DOI:** 10.1186/s13059-025-03681-9

**Published:** 2025-07-17

**Authors:** Philine Guckelberger, Leah Haut, Rosaria Tornisiello, Helene Kretzmer, Alexander Meissner

**Affiliations:** 1 Max Planck Institute for Molecular Genetics, Berlin, Germany; 2 Department of Biology, Chemistry and Pharmacy, Freie Universität Berlin, Berlin, Germany; 3 Current Address: Digital Health Cluster, Digital Engineering Faculty, Hasso Plattner Institute for Digital Engineering, University of Potsdam, Potsdam, Germany

**Keywords:** DNA methylation, Chromatin remodeling, Centromeres, Satellite repeats, HELLS, T2T

## Abstract

DNA methylation regulation involves multi-layered chromatin interactions that require remodeling proteins like the helicase, lymphoid-specific (HELLS). Here, we generate HELLS and DNA methyltransferase 3A and B (DNMT3A/B) knockout human pluripotent stem cells and report telomere-to-telomere maps of whole genome bisulfite sequencing data combined with ATAC-sequencing. Disrupting HELLS induces a global loss of DNA methylation that is distinct from the DNMTs, in particular over peri/centromeric satellite repeats as defined in the telomere-to-telomere genome assembly. However, HELLS appears dispensable for local enhancer remodeling and the potential to differentiate into the three embryonic germ layers. Taken together, our results further clarify the genomic targets and role of HELLS in human cells.

**Supplementary Information:**

The online version contains supplementary material available at 10.1186/s13059-025-03681-9.

## Background

HELLS is a highly conserved member of the SNF2 helicase family of chromatin remodelers [1–3], which are key players in regulating chromatin structure by using ATP hydrolysis to reposition, eject, or exchange nucleosomes [4, 5]. HELLS specifically has been implicated in de novo DNA methylation through its reported interactions with DNMT3A and DNMT3B [6–8]. Additionally, studies involving human cancer cell lines indicate a role for HELLS in DNA methylation maintenance through its interactions with UHRF1 at the replication fork [9]. In mice, the knockout of HELLS results in perinatal lethality with signs of reduced global DNA methylation levels [10–13], which has been suggested to impact genome integrity [14–17]. In vitro HELLS exhibits the ability to shift nucleosomes [18], thereby potentially facilitating DNA methyltransferase access to DNA [19]. While the impact of HELLS on DNA methylation has been explored in mice [20, 21], work in human cells has so far been limited to locus-specific array-based techniques or individual cytosine measurements by enzymatic restriction of bisulfite-converted DNA [9, 22]. HELLS mutations are found in immunodeficiency, centromeric region instability, facial anomalies (ICF) syndrome, and hypomethylation of specific centromeric satellite repeats on chromosomes 1, 6, and 19 has been observed [23]. Notably, centromere structures differ significantly between mice and humans: human centromeric and pericentromeric regions contain complex arrays of various satellite families, whereas mice primarily have simpler minor and major satellite repeats [24].

As part of the human telomere-to-telomere (T2T) genome, the structure of peri/centromeric satellite repeats (CenSat) is now available [25], which includes satellite-rich regions along with upstream and downstream sequences, such as the distal short arms of acrocentric chromosomes [26]. While not strictly pericentromeric, these regions predominantly consist of satellite repeat families enriched in peri/centromeric regions of other chromosomes. Satellite repeat families exhibit distinct characteristics and genomic distributions. Active alpha satellites (aSat), the only repeat class directly associated with functional centromeres, form higher-order repeats (HORs) of 171 bp tandem arrays and uniquely contribute to kinetochore formation and non-coding RNA transcription [26]. In contrast, all other satellite repeat classes are associated with pericentromeric regions [26]. Inactive alpha satellites, though structurally similar to active alpha satellites, lack kinetochore association and are often flanked by divergent or monomeric alpha satellite variants [26]. The T2T assembly also revealed large GC-rich beta and gamma satellite arrays; beta satellites (bSat) consist of 68 bp tandem monomers forming multimeric HORs, while gamma satellites (gSat) form clusters of 2–10 kb and consist of highly diverged 220 bp monomers [26, 27]. The T2T genome also provided further insights into the distribution of human satellites 1 A and 1B, which are highly AT-rich and form alternating 42 bp HORs [27]. Regions such as HSat1A were identified on chromosomes 3, 4, and 13, while HSat1B was mapped primarily to chromosome Y. Additionally, satellites 2 and 3 (HSat2 and HSat3), derived from pentameric repeats, form the largest satellite arrays in the human genome, particularly on chromosomes 1, 9, and 16 [26]. Finally, the T2T genome also includes previously unresolved satellite variants (“other centromeric satellites“) and transition regions, composed of duplications and sequences bridging satellite families, in total adding 1.2 Mb of new sequence data to the genome assembly [26]. Whole genome bisulfite sequencing (WGBS) when combined with this new T2T assembly offers a more powerful approach to study global DNA methylation patterns, including our ability to investigate previously unavailable genomic regions.

## Results and discussion

**Fig. 1:**
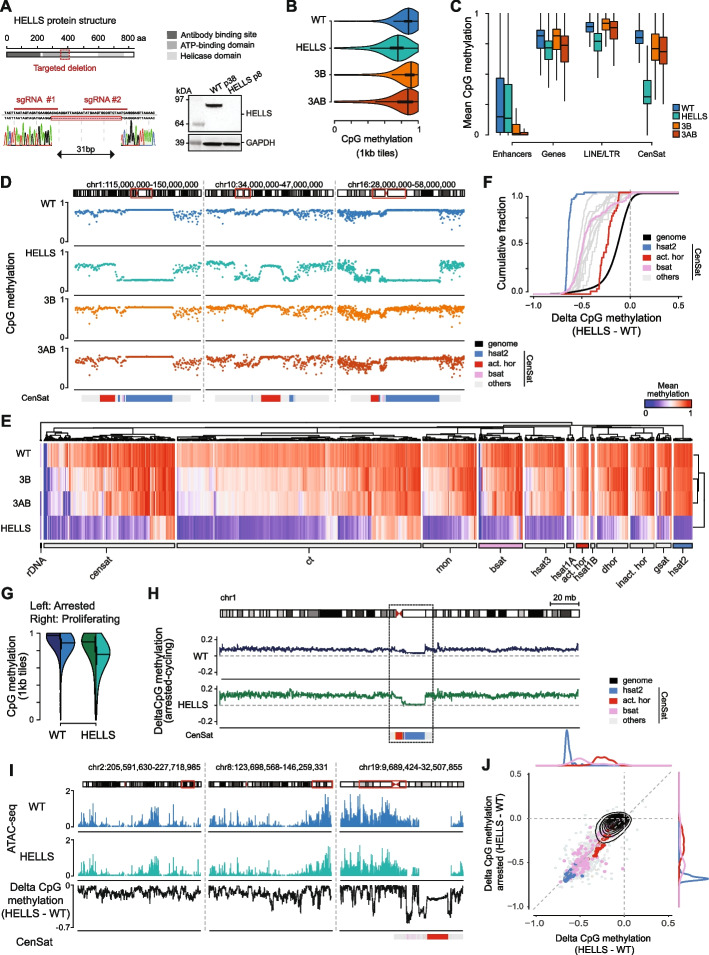
Human satellite repeats are key targets for HELLS activity. **A** Protein structure of HELLS including known functional domains and our guide RNA target sites. Below is the raw Sanger sequencing result of the homozygous HELLS KO iPSC clone (ZIP8K8, clone #B3) with a 31-bp deletion and the western blot verification. **B** Violin plots showing mean methylation over 1 kb tiles of whole genome bisulfite sequencing (WGBS) data generated for WT, the KO clones (HELLS: ZIP8K8, clone #B3, DNMT3B: ZIP34K14, clone #C1, and DNMT3A/B: ZIP34K14, clone #C3). Plots show median (horizontal line) and 25% and 75% quantiles (stronger and weaker vertical lines, respectively). *n* = 2,488,423 tiles. **C** Boxplots showing mean methylation over genomic features for WT and each of the knockout clones (HELLS, DNMT3B, and DNMT3A/B). The horizontal bar shows the median per feature, and boxes and whiskers reflect the quartiles. From left to right: n represents the number of regions included for calculating the DNA methylation distribution for each feature, with values of 16,201; 23,263; 1,411,943; 2,184. **D** Representative IGV browser tracks of the centromeric regions of three different chromosomes (1, 10, and 16) showing WGBS data for WT and the knockout clones (HELLS, DNMT3B, and DNMT3A/B) with satellite repeat class annotation (light gray), highlighting active alpha satellites (red), classical human satellite II (blue), and beta satellites (pink). **E** Heatmap visualizing the DNA methylation levels of the CenSat classes for WT and each of the knockout clones (HELLS, DNMT3B, and DNMT3A/B). The CenSat classes include: ribosomal DNA (rDNA), other centromeric satellites (censat), centromeric transition region (ct), monomeric alpha satellites (mon), beta satellites (bsat), classical human satellite III (hsat3), classical human satellite I type A (hsat1A), active alpha satellite (act. hor), classical human satellite I type B (hsat1B), divergent alpha satellite (dhor), inactive alpha satellite (incat. hor), gamma satellites (gsat), and classical human satellite II (hsat2). **F** Cumulative distribution plot of the delta DNA methylation between WT and the HELLS KO. The *y*-axis shows the cumulative fraction, representing the proportion of data points that are less than or equal to the corresponding value on the *x*-axis. **G** Split violin plots showing mean methylation over 1 kb tiles of WGBS generated for arrested (left half) and proliferating (right half) WT and HELLS KO. Plots show median (horizontal line) and 25% and 75% quantiles (stronger and weaker vertical lines, respectively). *n* = 2,488,423 tiles. **H** IGV browser track of chromosome 1 showing the change in DNA methylation between cycling as well as arrested WT and HELLS KO iPSCs. **I** Representative IGV browser tracks of loci across different chromosomes showing ATAC-seq for WT and HELLS KO and the change in DNA methylation between WT and HELLS KO. **J** Change in DNA methylation between arrested HELLS KO and WT cells as a function of change in DNA methylation between proliferating HELLS KO and WT cells

In light of these recent advances, we generated human HELLS knockout pluripotent stem cell lines to better understand the genomic targets of HELLS as well as its functional role. Given the prior literature, we were specifically interested in how HELLS affects genome-wide DNA methylation, particularly at centromeric and pericentromeric regions, which are crucial for genome stability but difficult to study due to their repetitive nature. In three independent targeting rounds of two induced pluripotent stem cell (IPSC) lines, we obtained a total of nine HELLS knockout clones that were validated by Sanger sequencing and western blot analysis (see the “ Methods” section; Fig. 1A, Additional file 1: Fig. S1A, B). FACS analysis confirmed that proliferation and cell-cycle distributions were unchanged in representative clones (Additional file 1: Fig. S1C). All HELLS knockout iPSC lines remained viable and continued to grow without any obvious phenotypic changes from 5 up to 33 passages (ZIP8K8, clone #B3). We selected ZIP8K8 (clone #B3) as well as one additional HELLS KO clone from each of the two different iPSC lines to ensure robustness of the results (ZIP8K8, clone #F11 and ZIP34K14, clone #G2; Additional file 1: Fig. S1B-D).

To better contextualize the genomic impact of HELLS disruption on DNA methylation, we also generated new single DNMT3B as well as double DNMT3A and DNMT3B knockout (DKO) lines (see “ Methods” section; Additional file 1: Fig. S1D). All samples, including early and late passages, were subjected to WGBS and aligned to the T2T reference genome (Fig. 1B–D, Additional file 1: Fig. S1E–H) [25]. The disruption of HELLS resulted in a modest genome-wide reduction in DNA methylation that affected 51.3% of CpGs and was largely stable over time (> 3 months in culture). Nonetheless, this effect far exceeds that of the de novo DNMT mutants, which show only 18–28% of all CpGs decreasing (Fig. 1B–D, Additional file 1: Fig. S1G, H, S2A). Notably, not all genomic regions responded equally across the knockouts, which allowed us to identify distinct preferences for genomic targets between HELLS and DNMT mutants. The DNMT mutants show slightly more pronounced effects on enhancers but weaker effects on gene bodies or dispersed repeats (Fig. 1C, Additional file 1: Fig. S2B, C). In contrast to the other regions, the HELLS mutant exhibits a unique methylation profile across the entire centromere and pericentromere, particularly at the satellite repeats (CenSat, Fig. 1C, Additional file 1: Fig. S2B–D) [25, 26]. This profile is markedly distinct from that of the DNMT mutants, which are more comparable to wild-type cells, with an average mean methylation loss of 0.11 for DKO and 0.42 for HELLS (Fig. 1D, E, Additional file 2: Table S1A). The decrease in methylation affects all classes of satellite repeats, though the extent of this effect varies in the HELLS knockout (Fig. 1D–F, Additional file 1: Fig. S2E–G). Active alpha satellite elements remain most highly methylated, while particularly class 2 satellite repeats (hsat2), which start from a high baseline methylation in WT (mean 0.88), exhibit the most pronounced change with a greater than 0.6 average loss in DNA methylation (Additional file 1: Fig. S2G). To explore the impact of sequence composition on delta methylation levels, we looked at repeat length, CpG count, CpG density, and GC content (excluding CpGs). GC content did not show any significant correlation, while length, CpG density, and CpG counts were associated (Additional file 1: Fig. S2H). However, a small subset of satellite repeats within almost every class—except for Class 2 and 3—showed significantly less methylation loss that could not be explained by the features we examined (Fig. 1E, Additional file 1: Fig. S2E). Of note, we excluded active alpha satellite sequences from this analysis due to their unique structural and functional properties, such as association with the kinetochore, which differentiate them from other satellite repeats. In addition to satellite repeats, several other genomic regions also exhibited significant DNA methylation loss in the absence of HELLS, including gene clusters and repetitive elements such as Olfactory Receptors (OR), Zinc Finger (ZNF), pseudogenes, and Immunoglobulin (IGHV) clusters (Additional file 1: Fig. S3A).

The substantial loss of methylation at centromeric regions specifically in the HELLS KO suggests difficulties with proper DNA methylation maintenance in this context. Although less striking, we had also observed a modest but genome-wide effect on DNA methylation in the knockout cells. Interestingly, DNA methylation in wildtype cells is also slightly below the theoretically expected levels. However, we have previously shown that arresting cells in the G2-M phase increases DNA methylation levels to near-completeness, as the arrest enriches for cells that have completed interphase, ensuring post-replication DNA methylation maintenance has occurred [28]. To determine whether a delay in cell cycle progression would also restore wildtype levels in the HELLS knockout cells, we treated both WT and HELLS KO iPSCs with nocodazole for 16 h to transiently arrest cells in G2-M phase [29] (Additional file 1: Fig. S3B, C). Interestingly, both the WT and HELLS KO cells exhibit a global increase in DNA methylation (WT median: 0.88 to 0.97 and HELLS KO median: 0.75 to 0.89, Additional file 2: Table S1A), although the HELLS KO remained at slightly lower levels (Fig. 1G, Additional file 1: Fig. S3D, E). However, the absolute and fractional increase in DNA methylation was overall higher in the arrested HELLS KO compared to the arrested WT cells (Fig. 1H, Additional file 1: Fig. S3F). Additionally, we observed a subset of CpGs that exhibited a failure to increase to WT levels, indicating a stronger dependence on HELLS for methylation (Additional file 1: Fig. S3D–F). Most strikingly, the low levels of DNA methylation at centromeric satellites persist even in the arrested HELLS KO (median < 0.4, Fig. 1J, Additional file 1: Fig. S3F, G, Additional file 2: Table S1B), thus distinguishing them from both other non-satellite repetitive regions and from other late-replicating regions.

Mechanistically, this pronounced effect on DNA methylation may be linked to reduced chromatin accessibility. Satellite repeats are typically located in more condensed, constitutive heterochromatin with high nucleosome density [24], which could potentially limit the accessibility to the DNA for DNMTs [19, 24]. To investigate this, we examined ATAC-seq signals across 5 kb genomic tiles and compared these patterns with changes in DNA methylation upon HELLS loss. Regions with lower baseline ATAC-seq signals, indicative of more compact chromatin, generally exhibited greater reductions in methylation (Pearson’s *R* =  − 0.6, Additional file 1: Fig. S4A). While ATAC-seq signal intensity and peak distribution were largely similar between WT and HELLS KO samples, we observed a distinct pattern in the centromeric transition zone of chromosome 19 (Fig. 1I). In this region, we detected a marked loss of ATAC-seq signal in the HELLS KO compared to WT. This reduction in chromatin accessibility coincided with a significant decrease in DNA methylation, suggesting a potential link between HELLS-mediated nucleosome remodeling and DNA methylation maintenance in this specific genomic context. Moreover, we found that gene clusters showing similar levels of DNA methylation loss in the absence of HELLS were frequently marked by inaccessible chromatin (Additional file 1: Fig. S3A).

Given the observed interplay between chromatin accessibility and methylation loss, we next examined whether these changes influenced transcriptional activity. Despite strong demethylation at satellite repeats, we did not observe major changes in repeat expression (Additional file 1: Fig. S4B, Additional file 2: Table S2A). Genome-wide gene expression analysis revealed a subset of dysregulated genes (Additional file 1: Fig. S4B, Additional file 2: Table S2B), although most of these transcriptional changes showed little association with methylation loss (Additional file 1: Fig. S5). Interestingly, a cluster of ZNFs (e.g., ZNF208, ZNF676, ZNF492, ZNF729, and ZNF257) located on chromosome 19 showed a strong decrease in DNA methylation but also a reduction in chromatin accessibility and expression (Fig. 1I, Additional file 1: Fig. S5). With a few exceptions, HELLS therefore seems generally more relevant for shaping global DNA methylation, and its impact on gene expression appears limited and possibly more indirect [30].

**Fig. 2:**
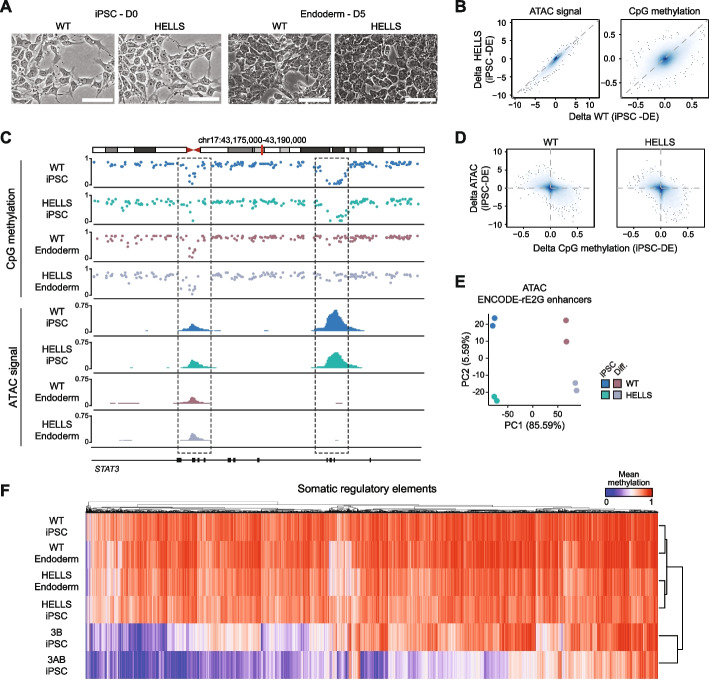
Enhancer elements do not require HELLS for differentiation-associated chromatin dynamics and DNA methylation regulation. **A** Directed differentiation of WT and HELLS KO iPSCs into endoderm (day 5, D5) imaged by brightfield at 40 ×. The white scale bar reflects 25 µm. **B** Smooth scatter plots comparing WT and HELLS KO delta iPSC and endoderm ATAC-seq signal (left panel) and CpG methylation (right panel) across all identified ATAC-seq peaks. The color scale indicates the density of CpGs with blue representing higher density. **C** Representative IGV browser tracks of a locus highlighting one static and one dynamic region. Tracks show DNA methylation and ATAC-seq signal of undifferentiated iPSCs and differentiated endoderm cells (WT and HELLS KO). **D** Smooth scatter plots comparing delta iPSC and endoderm ATAC-seq with delta iPSC and endoderm CpG methylation for WT (left panel) and HELLS KO (right panel) across all identified ATAC-seq peaks. The color scale indicates the density of CpGs with blue representing higher density. **E** PCA of ENCODE-rE2G enhancer predictions [34] in WT and HELLS KO iPSCs and endoderm (Diff.). **F** Heatmap visualizing the DNA methylation levels of putative somatic regulatory elements [35] for WT and each of the knockout clones (HELLS, DNMT3B, and DNMT3A/B iPSCs), as well as WT and HELLS KO endoderm

Since other chromatin remodelers in the same family have been implicated in enhancer regulation [31–33], we next focused on assessing the role of HELLS during differentiation. To first determine the general developmental potential of HELLS KO iPSCs, we derived cells of the three embryonic germ layers (endoderm, mesoderm, and ectoderm), as well as further differentiated pancreatic progenitor cells (see the “ Methods” section). Based on selected lineage markers, we did not identify any notable cellular deficiencies (Fig. 2A, Additional file 1: Fig. S6A–D). Since differentiation processes involve the remodeling of enhancer regions—both closing and opening—we performed a comparative analysis of chromatin accessibility and DNA methylation in undifferentiated as well as differentiated wild-type and HELLS knockout iPSCs using ATAC-seq and WGBS, respectively (Fig. 2B–E, Additional file 1: Fig. S6E–G). In line with the unperturbed differentiation, we observed a high correlation in ATAC-seq signal and methylation changes over enhancers [34] between WT and KO cells, indicating that HELLS is not required for remodeling at regulatory elements during the initial stages of lineage specification. Additionally, we observed consistent DNA methylation levels in HELLS KO cells at previously defined putative somatic regulatory elements [35] (Fig. 2F). These elements are characterized by the dynamic interplay between DNA methylation turnover (continuous addition and removal) in pluripotent cells, and distinguished by reduced methylation in DNMT3 DKOs [35, 36] (Fig. 2F). The similarity between the DNA methylation changes observed in WT and HELLS KO iPSCs and those observed in other features supports the observation of retained differentiation potential and suggests that HELLS does not play a role in DNMT3-mediated priming of somatic enhancers.

## Conclusions

Our results show that HELLS plays a limited or redundant role in gene regulatory elements. On the other hand, we find that the impact of HELLS depletion is more widespread than anticipated and not equally distributed across the genome. Specifically, HELLS appears crucial in regions where DNA methylation maintenance is more complex, such as late-replicating peri/centromeric satellite regions. HELLS may be needed in these areas to facilitate access of the DNMTs (DNMT1, DNMT3A and DNMT3B) to the DNA. Many details of satellite repeat subclasses were largely missing from the literature until the recent completion of the T2T genome, which provided us with the unique opportunity to map the specific molecular effects of HELLS depletion. Despite the inherent limitations of short reads, which still produce multimappers, restricting our analysis to unique mappers reveals the same molecular effects (Fig. 1C, E, Additional file 1: Fig. S2B, E). This suggests that the T2T genome already enables enhanced resolution and insights into repetitive regions, even without long-read sequencing.

Interestingly, a subset of satellite repeats appeared largely HELLS independent, which may reflect the rapid evolution of satellite repeat classes resulting in lower CpG density and differential regulation [37, 38]. Previous studies have suggested that different satellite repeat classes and families exhibit distinct accessibility profiles [39], which could contribute to the observed variations in HELLS dependency. Unfortunately, a more detailed assessment of nucleosome density effects is difficult with ATAC-seq due to its preference for nucleosome-free regions, which could mask more subtle differences within highly compact regions. While our data are generally consistent with previous results of satellite repeat methylation defects in ICF patients [9, 22], they also highlight the limitations of earlier techniques, such as limited arrays or restriction-based DNA methylation assessments, where entire satellite repeat classes are classified as hypomethylated based on individual cytosine measurements. Our approach and results provide a more detailed picture of HELLS and its impact on genome-wide DNA methylation. It is noteworthy that satellite repeats are dysregulated in various disease contexts [17, 40–43], including cancer and ICF syndrome. Consistent with the immunodeficiency observed in ICF patients, immune class switching—a process reliant on NHEJ—is impaired in the absence of HELLS [44]. This defect has been attributed to altered DDR signaling in HELLS-deficient cells, which is mechanistically interesting given our data that show extensive hypomethylation of IGHV genes (Additional file 1: Fig. S3A). Additionally, during the reprogramming of fibroblasts from ICF patients with DNMT3B mutations, methylation defects were observed in regions outside of satellite repeats [45, 46]. Together, this further highlights the possibility that disease-specific phenotypes may arise from HELLS deregulation that go beyond the alterations observed in the human pluripotent cells.

## Methods

### iPSC cell culture

We used two male iPSC lines for the generation of knockout clones. ZIP8K8 was previously reported [47, 48] and ZIP34K14 was derived from the same male donor fibroblasts. Both ZIP8K8 and ZIP34K14 (wildtype and mutant) iPSCs were maintained in mTeSR Plus (Stemcell Technologies, #100-0276) supplemented with 100 U/ml Penicillin and 100 µg/ml Streptomycin (Thermo Fisher Scientific, #15140122) on pre-coated culture ware (1:100 diluted Matrigel (Corning, #354234) in KnockOut DMEM (Thermo Fisher Scientific, #10829-018)) at 20–80% confluency by splitting every 2 to 3 days. Clump-based cell splitting was performed by incubating the cells in EDTA pH 8.0 (Thermo Fisher Scientific, #15575–038) and DPBS (Thermo Fisher Scientific, #14190-250) in a final concentration of 5 mM for 3 min at 37 °C and 5% CO_2_. Single-cell splitting was performed by incubating the cells with Accutase (Sigma-Aldrich, A6964) supplemented with 10 µM Y-27632 (Tocris, #1254) for 15 min at 37 °C and 5% CO_2_. All centrifugation steps were performed at 300 × g for 5 min at room temperature (RT) unless otherwise specified. Cell counting was performed using an automated cell counter (Thermo Fisher Scientific) on a single-cell suspension diluted 1:1 in 0.4% Trypan Blue staining solution (Thermo Fisher Scientific, #15250-061). Cells were routinely tested for Mycoplasma contamination using a PCR-based Mycoplasma Test Kit (AppliChem, #A8994).

Brightfield images were captured using a Nikon ECLIPSE Ts2 microscope with NIS-Elements software, and magnification details are specified in the figure legends.

### Genetic manipulation of iPSCs

For Cas9-based targeting we used ChopChop to design two exon-targeting single guide RNAs (sgRNAs, see Additional file 2: Table S3A for sequences) [49]. We cloned the sgRNAs in one expression vector system (Addgene plasmid #172221 after exchanging the GFP with the Puromycin resistance cassette from Addgene plasmid #62988) using the NEBuilder HiFi DNA Assembly Master Mix (#E2621S) according to the manufacturer’s instructions. The restriction enzymes BbsI (NEB) and SapI (NEB) were used to linearize the plasmid for cloning in the first sgRNA followed by the second sgRNA, respectively. We chemically transformed the assembled product into competent cells (Dh5a) and grew the bacteria on LB plates at 37 °C overnight. We selected single colonies to inoculate in liquid culture overnight, prepared the DNA (Qiagen, #27106X4) and used Sanger sequencing to verify successful cloning (see Additional file 2: Table S3B for sequences).

For the HELLS knockout, ZIP8K8 or ZIP34K14 iPSC single-cell suspensions were counted and seeded at a density of 1 × 10^6^ cells per 6-well in mTeSR Plus supplemented with a final 10 µM Y-27632. Cells were pre-cultured for 24 h at 37 °C and 5% CO_2_ prior to transfection. Cells were then transfected with 6 µg/6-well of the sgRNA containing P2X459 vector using Lipofectamin Stem Transfection Reagent (Thermo Fisher Scientific, #STEM00003) according to the manufacturer’s instructions. 24 h post-transfection, the cells were selected with 0.2 µg/ml Puromycin (Thermo Fisher Scientific, #A1113803) for 48-72 h. Single-cell derived colonies were manually picked and split two ways. Two-thirds of the colony was transferred to a 96-well for expansion, and the remaining third was used for genotyping using the Phire Animal Tissue Direct PCR Kit (Thermo Fisher Scientific, #F140WH) following the manufacturer’s instructions. Genotyping primers were designed such that both the WT and deletion band would be amplified (see Additional file 2: Table S3B for sequences). The PCR products of clones with an amplification band of decreased length compared to the WT were sent for Sanger sequencing to verify homozygous editing.

The DNMT3A as well as DNMT3A and 3B double-knockout were generated in ZIP34K14 in a similar fashion. The DKO were derived by targeting of DNMT3B in a DNMT3A only knockout cell line after seven passages.

### Western blot

iPSC single cell suspensions were washed once with ice cold DPBS and spun down at 300 × g, 5 min at 4 °C. Supernatants were removed and cells were resuspended in Triton Extraction Buffer (TEB: DPBS containing 0.5% Triton X-100 (v/v) and 1 × Protease inhibitor) and lysed for 10 min on ice with gentle stirring. The lysates were spun for 10 min at 6500* g* and 4 °C to pellet the nuclei. Nuclei were washed once with TEB to remove cell debris and again spun for 10 min at 6500* g* and 4 °C. Nuclei were then resuspended in 0.2 N HCl and incubated overnight at 4 °C. The next day, samples were spun for 10 min at 6500* g* and 4 °C to pellet the debris. The supernatant was transferred to a new tube and neutralized with 2 M NaOH at 1/10 of the supernatant volume. Reducing agent (Invitrogen, #NP0004), 40 mM Tris/Cl (pH 7.5) and Novex Tricine SDS Sample Buffer (2X) (Thermo Fisher Scientific, #LC1676) were added to the lysates, and the mixture was denatured at 85 °C for 2 min. Lysates were run on Novex 10 bis 20%, Tricin gels (Thermo Fisher Scientific, #EC6625BOX). Blots were transferred using the iBlot 2 Dry Blotting system with iBlot 2 transfer stacks (Thermo Fisher Scientific, #IB24001) and imaged by HRP chemiluminescence using SuperSignal West Dura Extended Duration Substrate (Thermo Fisher Scientific, #34075) and ChemiDoc XRS + System (Bio-Rad, #1708265). Western blots were performed using an anti-HELLS antibody (Santa Cruz, #sc-46665) at a 1:2000 dilution, an anti-DNMT3A antibody (Abcam, #ab188470) at a 1:2000 dilution, an anti-DNMT3B antibody (Cell Signaling Technologies, #67259) at a 1:1000 dilution, and an anti-GAPDH antibody (Cell Signaling #2118S).

### Three germ-layer differentiation

Three germ layer differentiation was performed using the STEMdiff Trilineage Differentiation media (Stemcell Technologies, #05230). Single-cell suspensions of mTeSR Plus-maintained iPSCs were seeded into 6-well plates at the cell number recommended by the manufacturer’s instructions. Fresh STEMdiff Trilineage Differentiation media was given to the cells every 24 h during the duration of the differentiation protocol. Cells were then collected at the specified time points (day 5 for endoderm and mesoderm; day 7 for ectoderm) using a single-cell split prepared for FACS analysis.

### Pancreatic progenitor differentiation

Pancreatic progenitor differentiation was performed using the STEMdiff Pancreatic Progenitor Kit (Stemcell Technologies, #05120). Single-cell suspensions of mTeSR Plus-maintained iPSCs were seeded into 6-well plates at the cell number recommended by the manufacturer’s instructions. Cells were differentiated through four stages, starting with definitive endoderm, to primitive gut tube, to posterior foregut endoderm, and finally pancreatic progenitor cells by changing the media according to the manufacturer’s instructions. On day 14, cells were collected using a single-cell split prepared for FACS analysis.

### Staining for FACS analysis of differentiation efficiency

iPSC single-cell suspensions were quenched with DPBS-10% Fetal Bovine Serum (FBS, PAN Biotech, #P30-2602)). Next, cells were centrifuged at 4 °C. Cells were then resuspended in DPBS-10% FBS containing surface marker antibodies and incubated for 15 min at 4 °C in the dark. Next, cells were washed once again with DPBS-10% FBS and centrifuged at 4 °C before FACS. Finally, cells were resuspended in DPBS-10% FBS and passed through a 70-µm cell strainer (Corning, #431751). FACS analysis was performed on the BD FACSAria Fusion Flow Cytometer.

An anti-NCAM antibody (Biolegend, #304608) was used at a 1:200 dilution for staining ectoderm and mesoderm; an anti-CXCR4 antibody (Biologend, #306506) was used at a 1:200 dilution for staining endoderm; and an anti-Glycoprotein 2 antibody (MBL International, #D277-5) was used at a 1:50 dilution for staining pancreatic progenitors (Additional file 1: Fig. S4).

Raw data were analyzed using FlowJo (LLC) V10.6.2.

### RNA isolation, cDNA synthesis, and quantitative PCR (qPCR)

We extracted RNA from 50,000 cells per experiment in Buffer RLT (Qiagen, #79216) using Dynabeads MyOne Silane beads (Thermo Fisher Scientific, #37002D), treated samples with TURBO DNase (Thermo Fisher Scientific, # AM2238), and cleaned again with Dynabeads MyOne Silane beads. We used AffinityScript reverse transcriptase (Agilent Technologies, #200436) and random hexamer primers (Therrmo Fisher Scientific, #SO142) to convert RNA to cDNA. We performed qPCR using SYBR Green I Master Mix (Roche, #04707516001) and calculated differences using the ΔΔCT method versus GAPDH (see Additional file 2: Table S3C for primer sequences). To achieve power to detect small effects in gene expression, we performed 3 technical qPCR replicates (from the same cDNA) and took the median value for further analysis. We also included three biological replicates.

### Cell cycle analysis and arrest

For the cell cycle analysis, cells were collected using a single-cell split and quenched with DPBS. 1 M cells were transferred into a protein lo-bind Eppendorf tube (#) and centrifuged.

The supernatant was discarded, and the cell pellet resuspended in 100 µl PBS-T (0.2% Tween 20, Thermo Fisher Scientific, #J20605.AP) To permeabilize and fix the cells 1 ml of 70% EtOH was added dropwise for a final concentration 1 ml/1 M cells and incubated at 4 °C for 2 h or overnight. For one wash, the cell suspension was then centrifuged and resuspended in 1 ml PBS-T. After centrifuging, the cell pellet was in 1 ml Dapi solution of 0.1% Triton-PBS (Triton-X-100. Thermo Fisher Scientific, #85111) and 1 µg/ml Dapi (Invitrogen, #D1306) and incubated at RT for 30 min in the dark. The cells were washed with 1 ml PBS-T. Finally, the cells were resuspended in 200 µl DPBS-10% FBS and filtered for FACS analysis. Cell cycle phase quantification was performed using the Dean-Jett-Fox model in FlowJo v10.

For the cell cycle arrest, cells were treated with 2 µg/ml nocodazole (Sigma-Aldrich, #M1404-10MG) for 16 h. The cells were harvested using a single-cell suspension. 1 M cells were taken for cell cycle analysis to verify the arrest, and the remaining were centrifuged, and the pellets snap-frozen using dry ice and stored at − 20 °C until further use.

### Whole genome bisulfite sequencing

Genomic DNA was extracted using Phenol–chloroform purification. In brief, cell pellets were resuspended in 200 µl DPBS, and 200 µl cold phenol–chloroform was added (1:1 ratio). After mixing by vortexing, the cell solution was centrifuged for 10 min at full speed. The aqueous layer was transferred to a fresh DNA-lo bind Eppendorf tube and supplemented with 10 µl of 5 M NaOH, 2 µl glycogen, and 500 µl 100% EtOH. After gentle vortexing, the solution was incubated at − 20 °C overnight. The next day, ethanol precipitation was performed by centrifuging the cell solution at 4 °C for 1 h at full speed. After discarding the supernatant, the pellet was resuspended in 1 ml of cold 70% EtOH and centrifuged again at 4 °C for 1 h at full speed. Finally, after discarding the supernatant and drying the pellet for 10 min, the DNA was resuspended in 25 µl of low EDTA TE buffer (Swift Biosciences, #90296). For the sample preparation, 300 ng genomic DNA was diluted in 50 µl low EDTA TE buffer and transferred to a Covaris tube (#520045) for fragmentation.

Next the DNA was fragmented using the following sonication settings: 10% cycle, PIP of 175, and 200 bursts/cycle for 2 × 45 s. The fragmented DNA was then transferred to a DNA lo-bind tube and purified using the Zymo Clean & Concentrator 5 Kit (#D4013) following the manufacturer’s instructions for fragmented DNA. The final product was eluted in 20 µl low EDTA TE buffer.

The purified genomic DNA was then bisulfite converted using the Zymo EZ DNA Methylation-Gold Kit (#D5005) by following the manufacturer’s instructions. The final product was eluted in 15 µl low EDTA TE buffer.

Finally, the sequencing library was prepared using Accel-NGS Methyl-seq DNA library kit (Swift Biosciences, #30024-SWI). The libraries were purified using Agencourt AMPure XP beads (Beckman Coulter, #A63881), and diluted to 4 nM for 150 bp paired-end sequencing on a NovaSeq 6000 platform (Illumina).

DNA collection for the early passage mutants occurred at passage 4 for the HELLS KO clone #B3, passage 5 for the HELLS KO clones #F11 and #G2, and passage 10 for DNMT3B and DNMT3AB knockouts. DNA collection for the late passage mutants occurred at passage 33 for the HELLS KO clone #B3 and passage 35 for DNMT3B and DNMT3AB knockouts. The WT control was collected at passage 34 and 63 for early and late passages, respectively.

### ATAC-seq

Two replicates each of undifferentiated iPSCs and endoderm-differentiated cells were harvested using a single-cell suspension and prepared for FACS analysis. Cells were stained for endoderm surface markers, and 50,000 cells were collected by sorting.

ATAC-seq libraries were prepared as described previously [50] with minor adjustments. In brief, 50,000 cells were centrifuged at 4 °C for 5 min at 500 × g. The cell pellet was resuspended in 50 µl of a water-based buffer containing 10 mM TRIS–HCl pH 7.4 (Thermo Fisher Scientific, #J67501-AK), 10 mM NaCl (Thermo Fisher Scientific, #AM9759), 3 mM MgCl_2_ (#AM9530G), 0.1% Igepal (Sigma-Aldrich, #I8896) and 0.1% Tween-20. To lyse the cells, the solution was incubated on ice for 3 min, after which the reaction was quenched with 1 ml of the resuspension buffer without lysing reagent (10 mM Tris–HCl pH 7.4, 10 mM NaCl, 3 mM MgCl_2_, and 0.1% Tween-20). Nuclei were collected by centrifuging the solution at 4 °C for 10 min at 500 × g. The supernatant was carefully removed, and the nuclei were resuspended in 50 µl of the DPBS-based transposition mixture consisting of a 2 × water-based buffer with 20 mM Tris–HCl pH 7.4, 10 mM MgCl_2_, 20% Dimethyl Formamide (Sigma-Aldrich, #PHR1553), and 0.1% Tween-20, 1% H_2_O, and 100 mM Transposase (EpiCypher, #15–1017). The transposition reaction was incubated in a thermomixer at 37 °C for 30 min at 1000 RPM. The reaction was purified using the Zymo Clean & Concentrator 5 Kit following the manufacturer’s instructions for fragmented DNA and was eluted in 20 µl. The sequencing library was prepared by amplifying the 20 µl purified DNA with a total of 50 µM Nextera indexing primers using the NEB Q5 Hot Start High Fidelity 2 × Master Mix (#M0494L) for 5 min at 72 °C, 30 s at 98 °C, and 9 cycles of 30 s at 98 °C, 30 s at 63 °C, and 1 min at 72 °C, followed by cooling down to 4 °C.

The final libraries were purified using Agencourt AMPure XP beads and diluted to 4 nM for 100 bp paired-end sequencing on a NovaSeq 6000 platform.

### Total RNA sequencing

RNA was extracted from confluent 6-well plates using the RNeasy Plus Micro Kit (Qiagen, #74034). Three biological replicates were collected per condition. Each sample was resuspended in 350 µl of RLT buffer containing 10% β-mercaptoethanol (Thermo, #21985023*)*, followed by the addition of 350 µl of 70% ethanol. RNA was then purified according to the manufacturer’s instructions. RNA quality and concentration were assessed prior to library preparation. For RNA sequencing, the KAPA RNA HyperPrep Kit (Roche, # KK8560) was used to generate libraries from 500 ng RNA per sample. The final libraries were purified using Agencourt AMPure XP beads and diluted to 4 nM for 100 bp paired-end sequencing on Element Biosciences’ AVITI system.

### Whole genome bisulfite sequencing processing

Raw reads were subjected to adapter and quality trimming using cutadapt (version 2.4; parameters: --quality-cutoff 20 --overlap 5 --minimum-length 25; Illumina TruSeq adapter clipped from both reads), followed by trimming of 10 and 5 nucleotides from the 5′ and 3′ ends of the first read and 15 and 5 nucleotides from the 5′ and 3′ ends of the second read [51].The trimmed reads were aligned to the human genome (chm13) using BSMAP (version 2.90; parameters: -v 0.1 -s 16 -q 20 -w 100 -S 1 -u -R) [52]. Duplicates were removed using the “MarkDuplicates” command from GATK (version 4.1.4.1; --VALIDATION_STRINGENCY = LENIENT --REMOVE_DUPLICATES = true) [53]. Methylation rates were called using mcall from the MOABS package (version 1.3.2; default parameters) [54]. All analyses were restricted to autosomes, and only CpGs covered by at least 10 and at most 150 reads were considered for downstream analyses.

In addition, we first filtered the aligned BAM files for uniquely aligned reads (bitwise flag required to not include 256). These uniquely mapped reads BAM files were subjected to the same downstream processing, i.e., methylation rate calling using mcall and coverage filtering of 10–150 reads as well as reduction to autosomes, prior to downstream analysis. Median WGBS reads for each feature considering either multi- or unique-mapped reads are included in Additional file 2: Table S4.

### ATAC-seq processing

Raw reads were subjected to adapter and quality trimming with cutadapt (version 2.4; parameters: --quality-cutoff 20 --overlap 5 --minimum-length 25 --adapter AGATCGGAAGAGC -A AGATCGGAAGAGC) as were their respective input samples. Reads were aligned to the human genome (chm13) using BWA with the “mem” command (version 0.7.17, default parameters) [55]. A sorted BAM file was obtained and indexed using samtools with the “sort” and “index” commands (version 1.10) [56]. Duplicate reads were identified and removed using GATK (version 4.1.4.1) ‘MarkDuplicates’ and default parameters. After careful inspection and validation of high correlation, replicates of treatment and input samples were merged as FASTQ and reprocessed as a pool.

Peaks were called using MACS2 “callpeak” (version 2.1.2) based on merged replicates [57].

### RNA-seq processing

Raw reads were subjected to adapter and quality trimming with cutadapt (version 4.4; parameters: --quality-cutoff 20 --overlap 5 --minimum-length 25 --interleaved --adapter AGATCGGAAGAGC -A AGATCGGAAGAGC), followed by poly-A trimming with cutadapt (parameters: --interleaved --overlap 20 --minimum-length --adapter "A[100]" --adapter "T[100]"). Reads were aligned to the reference genome using STAR (version 2.7.9a; parameters: --runMode alignReads --chimSegmentMin 20 --outSAMstrandField intronMotif --quantMode GeneCounts) and transcripts were quantified using stringtie (version 2.0.6; parameters: -e) with GENCODE annotation.

### Analysis

#### Differential expression analysis

Differentially expressed genes and repeats in the HELLS KO RNA-seq samples compared to WT were evaluated using TEtranscripts [58] (version 2.2.3, parameters: --stranded reverse --sortByPos), and the T2T-CHM13v2.0 [25] as genome reference. The gene and repeats set of interest was selected according to the following criteria: (1) *p*-value < 0.05, (2) log2(fold-change) > 1 for upregulated targets, (3) log2(fold-change) < 1 for downregulated targets.

#### Feature annotation

One kb genomic tiles were generated by segmenting the genome using bedtools makewindows (version 2.30.0; parameters: -w 1000 -s 1000) [59]. Annotations of repeats and centromeric features were downloaded from UCSC [25]. Annotations of putative endoderm enhancers were downloaded from ENCODE [34]. Putative somatic enhancers were derived from Charlton et al. 2020 [35]. Average DNA methylation values across the genomic features were computed by using bedtools to intersect the tiles with the processed methylation bedgraph for each sample, followed by calculating the mean and median methylation within each feature using R (Additional file 2: Table S1A). The values of average DNA methylation were based on these methylation values, and changes in DNA methylation were calculated by subtracting the methylation values of different conditions or features.

#### Statistics and software

If not stated otherwise: All statistics and plots are generated using R version 4.2.2 (2022-10-31) – “Innocent and Trusting”. In all boxplots, the centerline is the median; boxes, first and third quartiles; whiskers, 1.5 × inter-quartile range; data beyond the end of the whiskers are displayed as points.

#### Smooth scatter plots

Genome-wide comparison of CpG methylation rates and ATAC signal as smooth scatter plots was done using the R package smoothScatter function, with data point density encoded by color.

#### Methylation distribution plots

Average CpG methylation over annotated features, i.e., enhancer, genes, repeats, and CenSats, was plotted using the boxplot function of the ggplot2 (v3.3.2) package [60]. For genome-wide methylation distributions, 1 kb average tile methylation values were used for visualization with the vioplot (v0.3.5) package [61].

#### Heatmaps

First, the average methylation rates within annotated regions of different types of CenSat elements were calculated (Additional file 2: Table S1B). The average methylation rates of each element and each sample were subsequently visualized using the Complex Heatmap package [62, 63].

#### Scatter and cumulative delta plots

The average methylation rates within annotated regions of different types of CenSat elements were first calculated, to then subtract the averages by genomic element to calculate the delta methylation. The delta values were visualized using the ecdf or density_2d function of the ggplot2 package [60].

#### Browser tracks

Browser track figures were created using IGV (v2.9.2) [64], with the mean function activated. Centromeric satellite annotation was loaded in addition for visualization of repeat classes.

## Supplementary Information

Additional file 1. Fig. S1: Characterization of HELLS KO and DNMT mutant DNA methylation landscapes. Fig. S2: Characterization of HELLS KO and DNMT mutant DNA methylation levels at satellite repeats. Fig. S3: HELLS is indispensable for maintenance of DNA methylation at satellite repeats. Fig. S4: Chromatin and transcriptional features of HELLS KO iPSCs. Fig. S5: Overview of delta DNA methylation between WT and HELLS KO across individual chromosomes. Fig. S6: HELLS is not required to remodel enhancer landscapes essential for early embryonic lineage formation. Fig. S7: Gating strategy for sorting three-germ layer differentiated human iPSCs.

Additional file 2. Table S1A: Mean feature methylation. Table S1B: CenSat CpG methylation. Table S2A: RNA Repeats. Table S2B: RNA genes. Table S3A: gRNAs. Table S3B: Sanger sequencing and genotyping. Table S3C: PCR primers.

Additional file 3.

Additional file 4.

## Data Availability

Human iPSC sequencing data sets have been deposited in the Gene Expression Omnibus and are accessible under GSE241688 (Ref [65]). Source data and code used in this study have been deposited at Zenodo [66].
