# Supplementary material for: HELLS is required for maintaining proper DNA modification at human satellite repeats
Source: Genome Biol. 2025 Jul 17;26:211. doi: 10.1186/s13059-025-03681-9 (PMC12273238; doi:10.1186/s13059-025-03681-9)
Supplement: Supplementary file 1 — Additional file 1. Fig. S1: Characterization of HELLS KO and DNMT mutant DNA methylation landscapes. Fig. S2: Characterization of HELLS KO and DNMT mutant DNA methylation levels at satellite repeats. Fig. S3: HELLS is indispensable for maintenance of DNA methylation at satellite repeats. Fig. S4: Chromatin and transcriptional features of HELLS KO iPSCs. Fig. S5: Overview of delta DNA methylation between WT and HELLS KO across individual chromosomes. Fig. S6: HELLS is not required to remodel enhancer landscapes essential for early embryonic lineage formation. Fig. S7: Gating strategy for sorting three-germ layer differentiated human iPSCs. [file 13059_2025_3681_MOESM1_ESM.docx]

# **Additional File 1, Figs. S1-7**


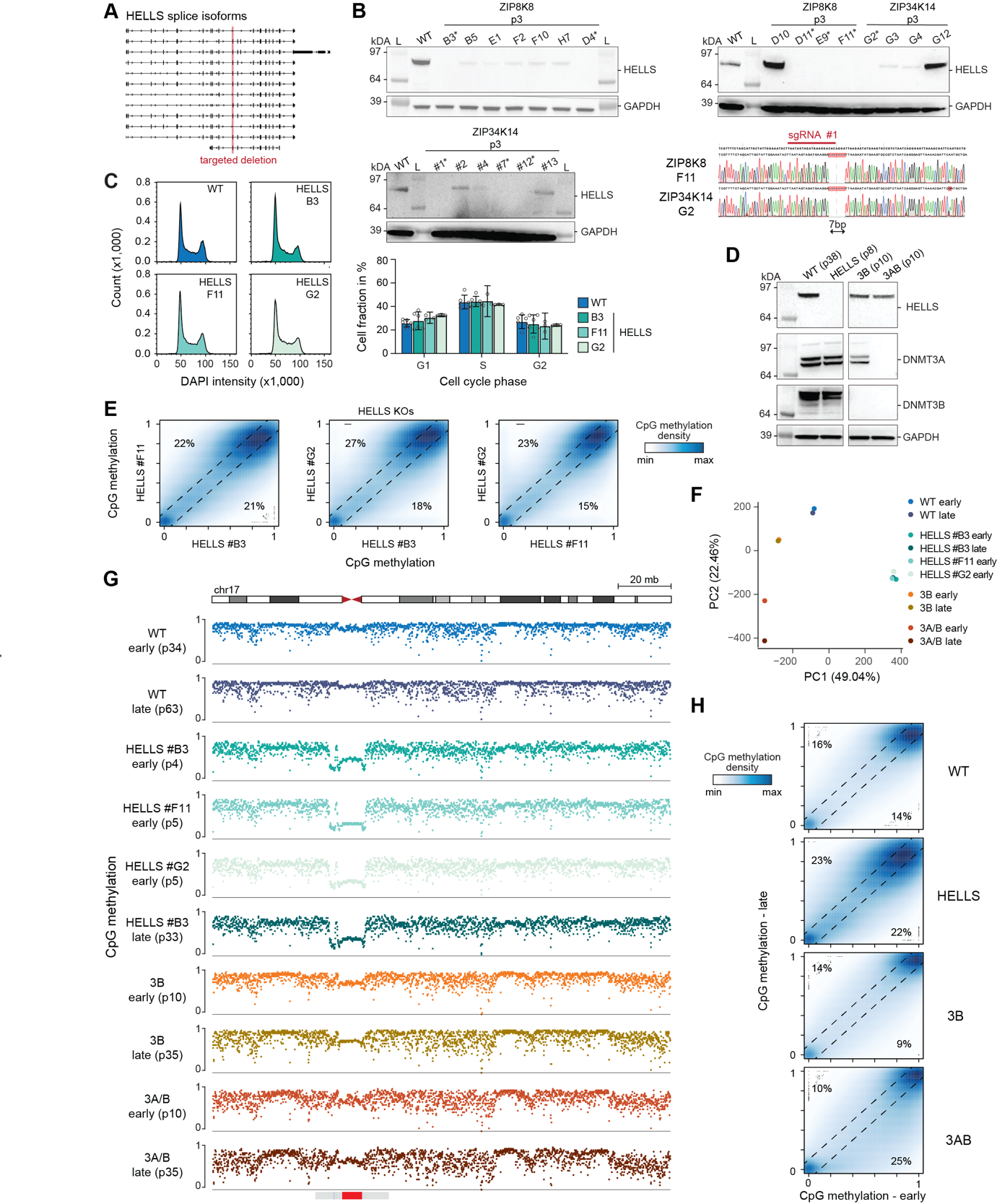


**Supplementary Figure 1. Characterization of HELLS KO and DNMT mutant DNA methylation landscapes**

**A)** Depiction of HELLS splice isoforms. The red highlight indicates the targeted deletion site of the gRNAs across the isoforms.

**B)** Western blot analysis of candidate HELLS KO clones, with successfully targeted clones marked by an asterisk and raw Sanger sequencing results of the additional homozygous HELLS KO iPSC clones used for validation (#F11 and #G2), each carrying a 7 bp deletion.

**C)** FACS-based cell cycle profiles of WT (blue) and three HELLS KO clones (green: #B3, #F11, and #G2) stained with DAPI. A representative cell cycle profile from one of the independent replicates is shown. The data include six independent replicates for WT and HELLS KO clone #B3, and three independent replicates for HELLS KO clones #F11 and #G2.

**D)** Western blot verifying the KO genotypes. The same samples were loaded and stained across gels and stained for either HELLS, DNMT3A, or DNMT3B. The lower half of each gel was cut off and stained for GAPDH as an internal control.

**E)** Smooth scatter plots comparing individual CpGs between the three HELLS KO clones (#B3, #F11, #G2). The x-axis and y-axis each represent the methylation level of the specified clone. The color scale indicates the density of CpGs at a particular methylation level, with blue representing higher density. The dotted lines represent a threshold of 0.1 change in methylation levels between conditions. The percentages indicate the proportion of CpGs with a greater than 0.1 difference in methylation between the respective clones.

**F)** PCA all early passage clones (WT p34, HELLS #B3 p4, HELLS #F11 p5, HELLS #G2 p5, 3B p10, 3A/B p10) and late passage clones (WT p63, HELLS #B3 p33, 3B p35, 3A/B p35).

**G**) IGV browser shot of the entire chromosome 17 showing DNA methylation (WGBS) of early passage clones (WT p34, HELLS #B3 p4, HELLS #F11 p5, HELLS #G2 p5, 3B p10, 3A/B p10) and late passage clones (WT p63, HELLS #B3 p33, 3B p35, 3A/B p35) with satellite repeat class annotation (light grey), highlighting active alpha satellites (red), classical human satellite II (blue), and beta satellites (pink).

**H)** Smooth scatter plots comparing individual CpGs between early (WT p34, HELLS #B3 p4, 3B p10, 3A/B p10) and late passage (WT p63, HELLS #B3 p33, 3B p35, 3A/B p35) clones, respectively. The x-axis represents the methylation level of CpGs in the early passage clones, while the y-axis represents the methylation level in the respective late passage clones. The color scale indicates the density of CpGs at a particular methylation level, with blue representing higher density. The dotted lines represent a threshold of 0.1 change in methylation levels between conditions. The percentages indicate the proportion of CpGs with a greater than 0.1 difference in methylation between the respective clones.


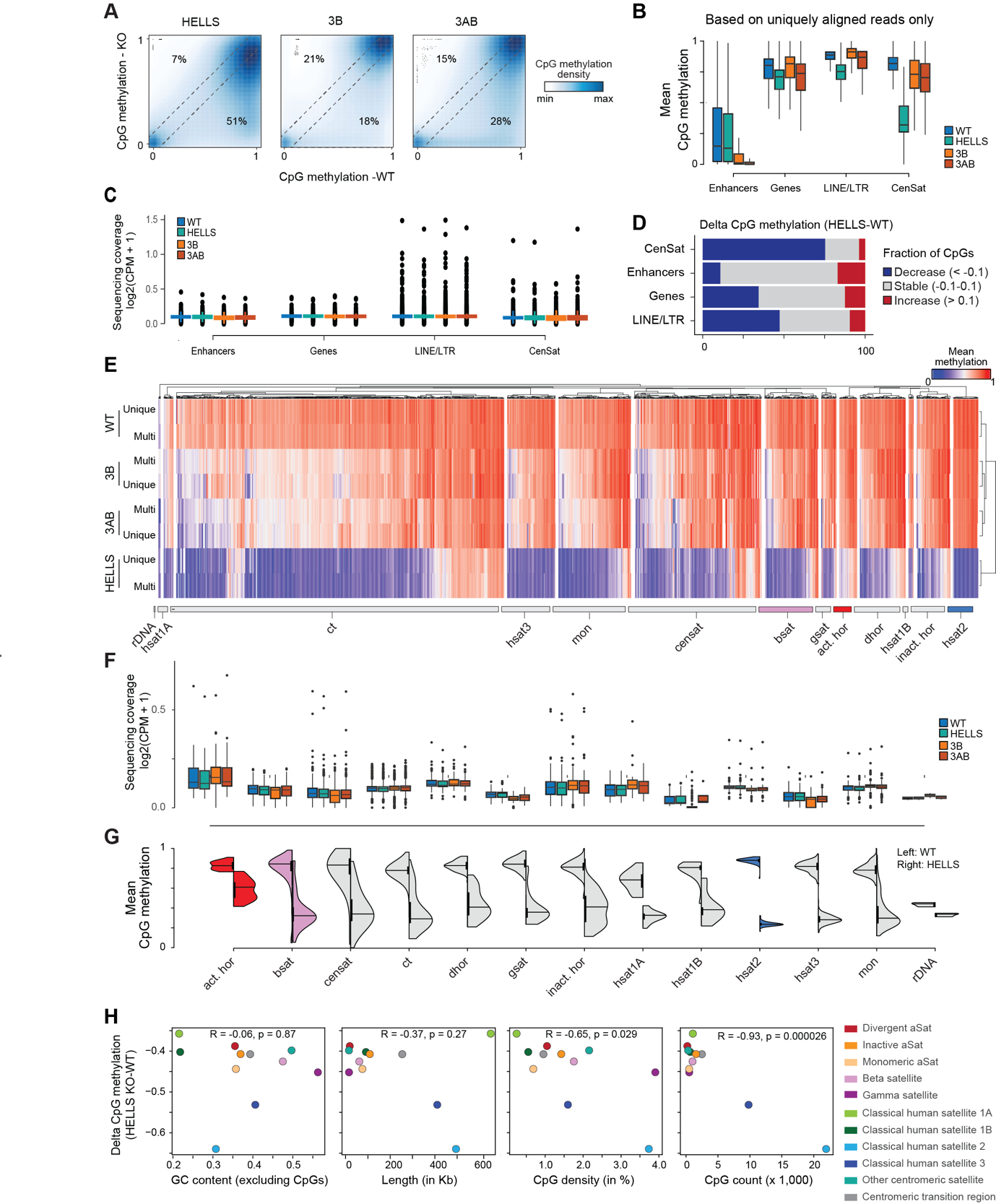


**Supplementary Figure 2. Characterization of HELLS KO and DNMT mutant DNA methylation levels at satellite repeats**

**A)** Smooth scatter plots comparing individual CpGs of all knockout clones (HELLS, 3B, 3AB) to WT. The x-axis represents the methylation level of CpGs in WT cells, while the y-axis represents the methylation level in the respective KO clones. The color scale indicates the density of CpGs at a particular methylation level, with blue representing higher density. The dotted lines represent a threshold of 0.1 change in methylation levels between conditions. The percentages indicate the proportion of CpGs with a greater than 0.1 difference in methylation between WT and the respective KO

**B)** Boxplots showing mean methylation over genomic features for WT and each of the knockout clones (HELLS, DNMT3B, and DNMT3A/B), considering only uniquely mapped regions. The horizontal bar shows the median per feature, and boxes and whiskers reflect the quartiles. From left to right: n represents the number of regions included for calculating the DNA methylation distribution for each feature, with values of 16,194; 23,164; 1,400,894; 2,076.

**C)** Boxplots showing WGBS read coverage over genomic features for WT and each of the knockout clones (HELLS, DNMT3B, and DNMT3A/B). The horizontal bar shows the median per feature, and boxes and whiskers reflect the quartiles. From left to right: n represents the number of regions included for calculating the coverage distribution for each feature, with values of 16,201; 23,263; 1,411,943; 2,184

**D**) Stacked bar plot visualizing the fraction of CpGs over genomic with DNA methylation level changes in the absence of HELLS compared to WT iPSCs. CpGs are categorized based on the magnitude of DNA methylation change in the absence of HELLS: increases (delta >0.1, red), stable (delta -0.1 -0.1, gray), decreases (delta <-0,1, blue).

**E)** Heatmap visualizing the DNA methylation levels of the CenSat classes for WT and each of the knockout clones (HELLS, DNMT3B, and DNMT3A/B), considering either all reads or only uniquely mapped reads. The CenSat classes include: ribosomal DNA (rDNA), other centromeric satellites (censat), centromeric transition region (ct), monomeric alpha satellites (mon), beta satellites (bsat), classical human satellite III (hsat3), classical human satellite I type A (hsat1A), active alpha satellite (act.hor), classical human satellite I type B (hsat1B), divergent alpha satellite (dhor), inactive alpha satellite (incαt.hor), gamma satellites (gsat), and classical human satellite II (hsat2).

**F)** Boxplots showing WGBS read coverage over satellite repeats classes for WT and each of the knockout clones (HELLS, DNMT3B, and DNMT3A/B). The horizontal bar shows the median per feature, and boxes and whiskers reflect the quartiles. The CenSat classes include: ribosomal DNA (rDNA), other centromeric satellites (censat), centromeric transition region (ct), monomeric alpha satellites (mon), beta satellites (bsat), classical human satellite III (hsat3), classical human satellite I type A (hsat1A), active alpha satellite (act.hor), classical human satellite I type B (hsat1B), divergent alpha satellite (dhor), inactive alpha satellite (incαt.hor), gamma satellites (gsat), and classical human satellite II (hsat2). with values of 41; 184; 662; 856; 103; 64; 79; 24; 64; 61; 189; 191; 5.

**G)** Split violin plots showing mean methylation over satellite repeat classes generated for WT (left) and HELLS knockout (right). Plots show median (horizontal line) and 25% and 75% quantiles (stronger and weaker vertical lines, respectively). The CenSat classes include: ribosomal DNA (rDNA), other centromeric satellites (censat), centromeric transition region (ct), monomeric alpha satellites (mon), beta satellites (bsat), classical human satellite III (hsat3), classical human satellite I type A (hsat1A), active alpha satellite (act.hor), classical human satellite I type B (hsat1B), divergent alpha satellite (dhor), inactive alpha satellite (incαt.hor), gamma satellites (gsat), and classical human satellite II (hsat2). The CenSat classes include: ribosomal DNA (rDNA), other centromeric satellites (censat), centromeric transition region (ct), monomeric alpha satellites (mon), beta satellites (bsat), classical human satellite III (hsat3), classical human satellite I type A (hsat1A), active alpha satellite (act.hor), classical human satellite I type B (hsat1B), divergent alpha satellite (dhor), inactive alpha satellite (incαt.hor), gamma satellites (gsat), and classical human satellite II (hsat2). From left to right: n represents the number of regions included for calculating the DNA methylation distribution for each satellite repeat class, with values of 41; 184; 662; 856; 103; 64; 79; 24; 64; 61; 189; 191; 5.

**H)** Scatterplot showing the relationship between delta DNA methylation between WT and HELLS KO and, from left to right, average GC content, element length, CpG density, and CpG count for CenSat repeat classes. The CenSat classes include: other centromeric satellites (censat), centromeric transition region (ct), monomeric alpha satellites (mon), beta satellites (bsat), classical human satellite III (hsat3), classical human satellite I type A (hsat1A), classical human satellite I type B (hsat1B), divergent alpha satellite (dhor), inactive alpha satellite (incαt.hor), gamma satellites (gsat), and classical human satellite II (hsat2).


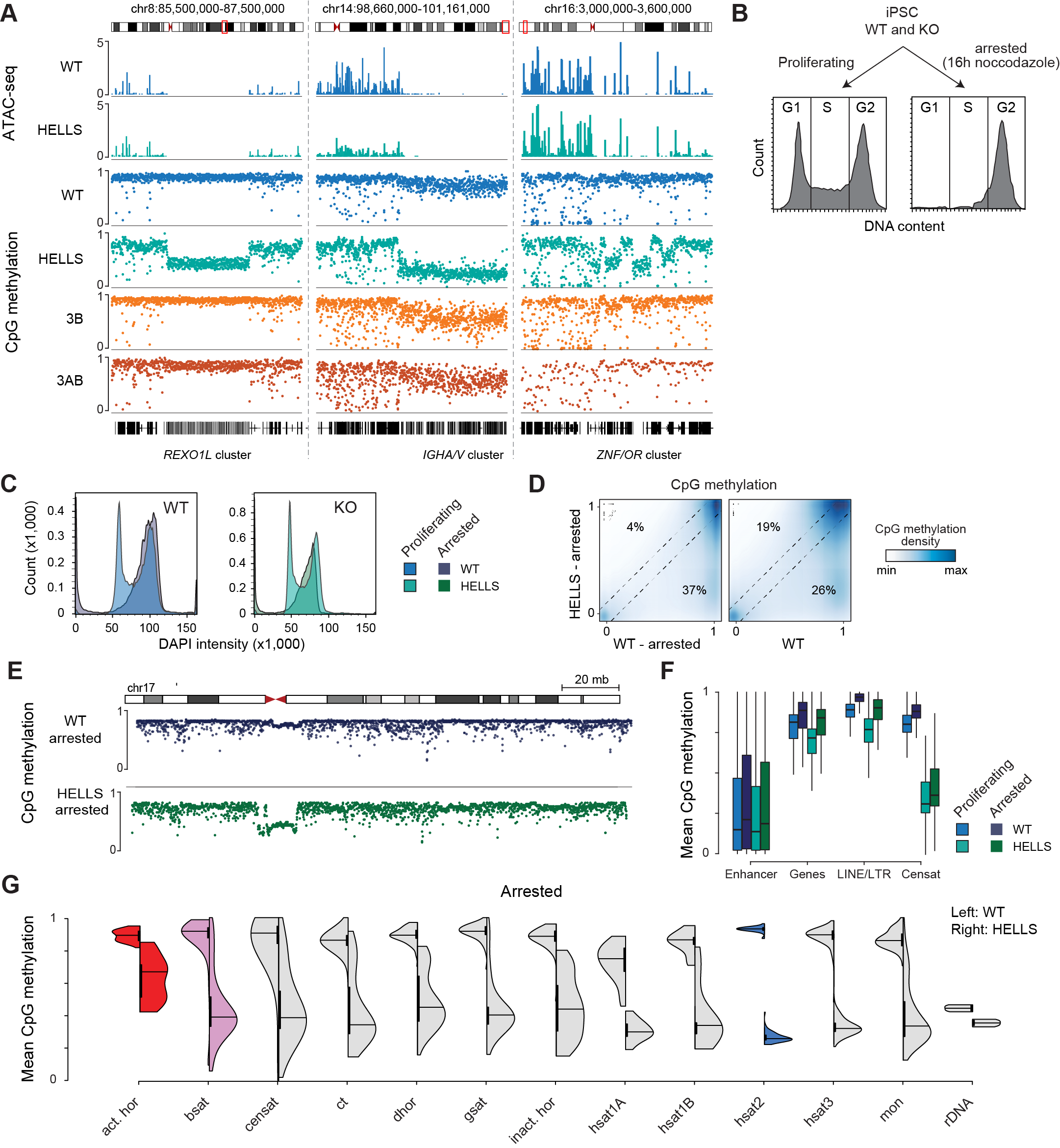


**Supplementary Figure 3. HELLS is indispensable for maintenance of DNA methylation at satellite repeats**

**A)** Representative IGV browser tracks of non-centromeric regions with pronounced effects of HELLS on DNA methylation on three different chromosomes (8, 14, and 16) showing WGBS data for WT and the knockout clones (HELLS, DNMT3B, and DNMT3A/B).

**B)** Schematic of experimental design. iPSCs were grown in proliferating culture conditions reflected in the representative FACS-based cell cycle profile (left panel) or in G2-arrested conditions by the presence of the mitotic inhibitor nocodazole for 16h reflected by the shift in the representative FACS blot to G2-M phase (4N) (right panel). Genomic DNA was extracted from both conditions, and WGBS was performed.

**C)** FACS-based cell cycle profiles of WT (blue, left panel) and HELLS knockout (green, right panel) proliferating (light hue) and G2-arrested (dark hue) cells stained with DAPI.

**D)** Smooth scatter plots comparing individual CpGs in WT arrested and proliferating HELLS arrested cells. The y-axis represents the methylation level of CpGs in HELLS arrested cells, while the x-axis represents the methylation level in WT arrested cells (left panel) and WT proliferating cells (right panel). The color scale indicates the density of CpGs at a particular methylation level, with blue representing higher density. The dotted lines represent a threshold of 0.1 change in methylation levels between conditions. The percentages indicate the proportion of CpGs with a greater than 0.1 difference in methylation between WT and the respective KO.

**E**) IGV browser shot of the entire chromosome 17 showing DNA methylation (WGBS) of arrested WT and HELLS KO.

**F)** Boxplots showing mean methylation over genomic features for proliferating and arrested WT and HELLS KO. The horizontal bar shows the median per feature, and boxes and whiskers reflect the quartiles. From left to right: n represents the number of regions included for calculating the DNA methylation distribution for each feature, with values of 16,201; 23,263; 1,411,943; 2,184.

**G)** Split violin plots showing mean methylation for WT (left) and HELLS (right) arrested iPSCs over satellite repeat classes. Plots show median (horizontal line) and 25% and 75% quantiles (stronger and weaker vertical lines, respectively). The CenSat classes include: ribosomal DNA (rDNA), other centromeric satellites (censat), centromeric transition region (ct), monomeric alpha satellites (mon), beta satellites (bsat), classical human satellite III (hsat3), classical human satellite I type A (hsat1A), active alpha satellite (act.hor), classical human satellite I type B (hsat1B), divergent alpha satellite (dhor), inactive alpha satellite (incαt.hor), gamma satellites (gsat), and classical human satellite II (hsat2). From left to right: n represents the number of regions included for calculating the DNA methylation distribution for each satellite repeat class, with values of 41; 184; 662; 856; 103; 64; 79; 24; 64; 61; 189; 191; 5.


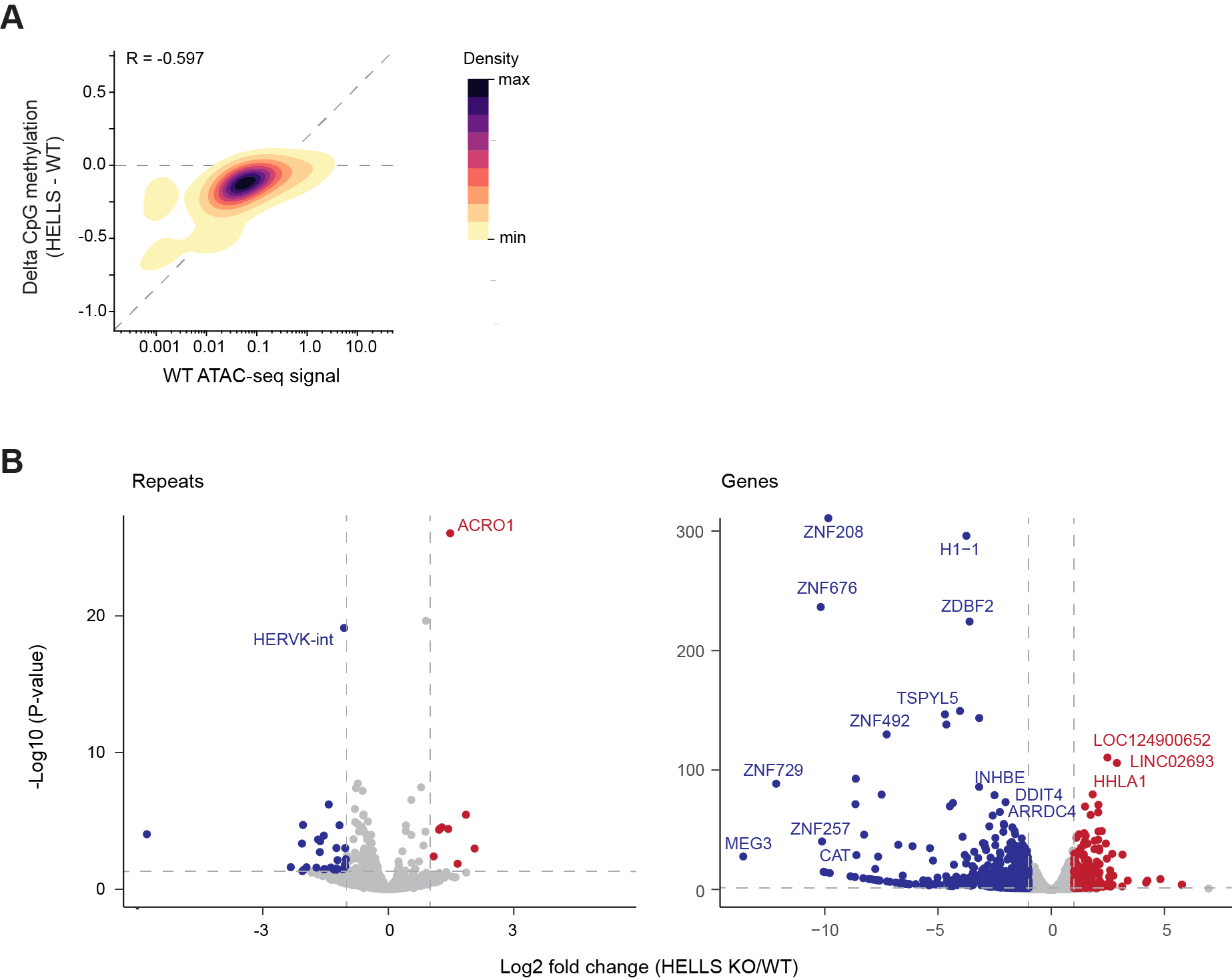


**Supplementary Figure 4. Chromatin and transcriptional features of HELLS KO iPSCs**

**A)** Kernel density estimate plot of WT ATAC signal as a function of delta DNA methylation between WT and HELLS KO clone #B3 across 5kb tiles.

**B)** Volcano plot highlighting repeat (left panel) and gene (right panel) expression differences between WT and HELLS KO iPSCs. Significantly down- and up-regulated elements with a log2FC< 1 or > 1 are illustrated as blue and red circles, respectively.


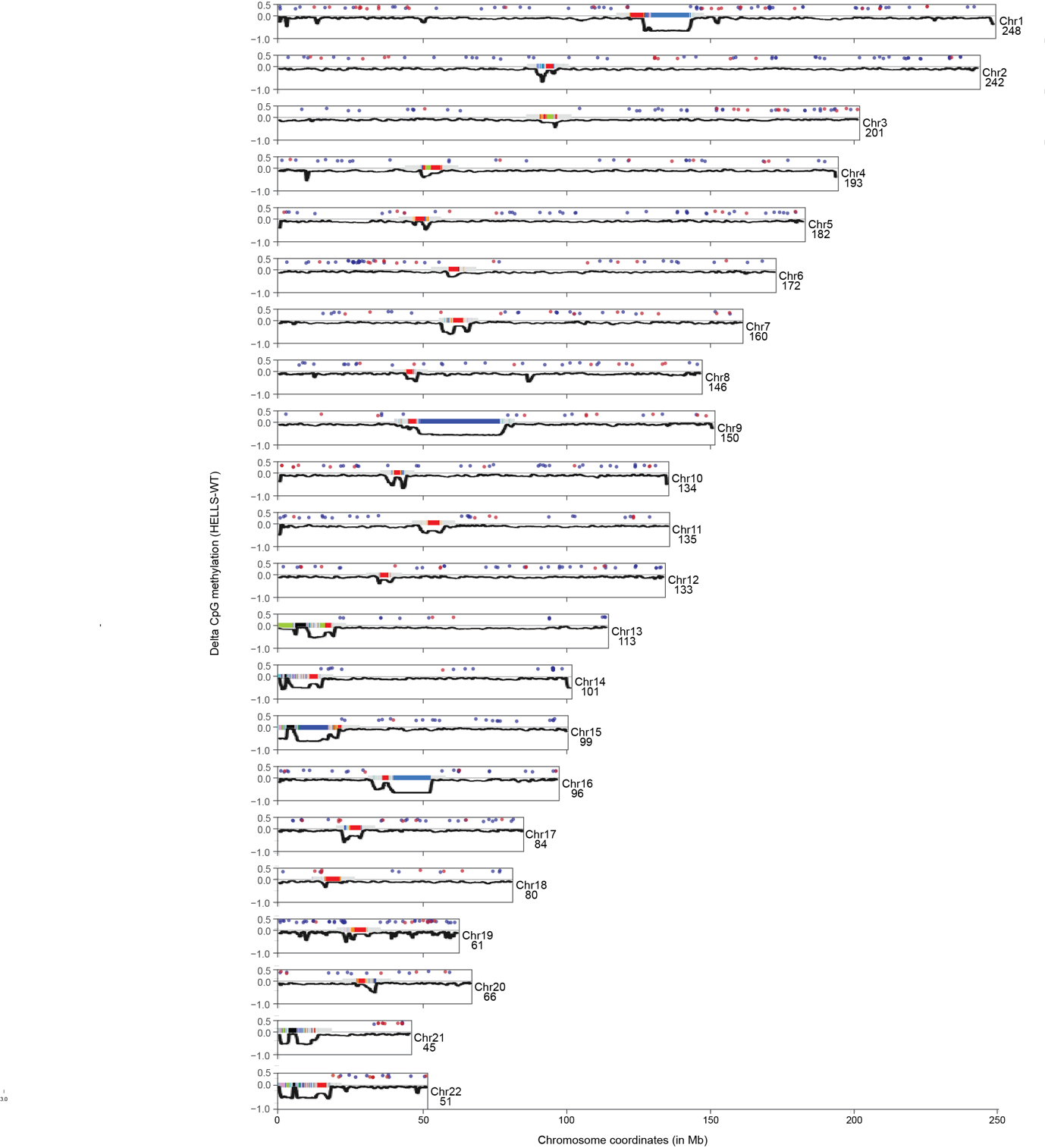


**Supplementary Figure 5. Overview of delta DNA methylation between WT and HELLS KO across individual chromosomes**

The CenSat repeats are colored by class: ribosomal DNA (black), other centromeric satellites (teal), centromeric transition region (gray), monomeric alpha satellites (peach), beta satellites (pink), classical human satellite III (blue), classical human satellite I type A (light green), active alpha satellite (red), classical human satellite I type B (dark green), divergent alpha satellite (dark red), inactive alpha satellite (orange), gamma satellites (purple), and classical human satellite II (light blue). Significantly down- and up-regulated genes with a log2FC< 1 or > 1 are illustrated as blue and red circles, respectively.


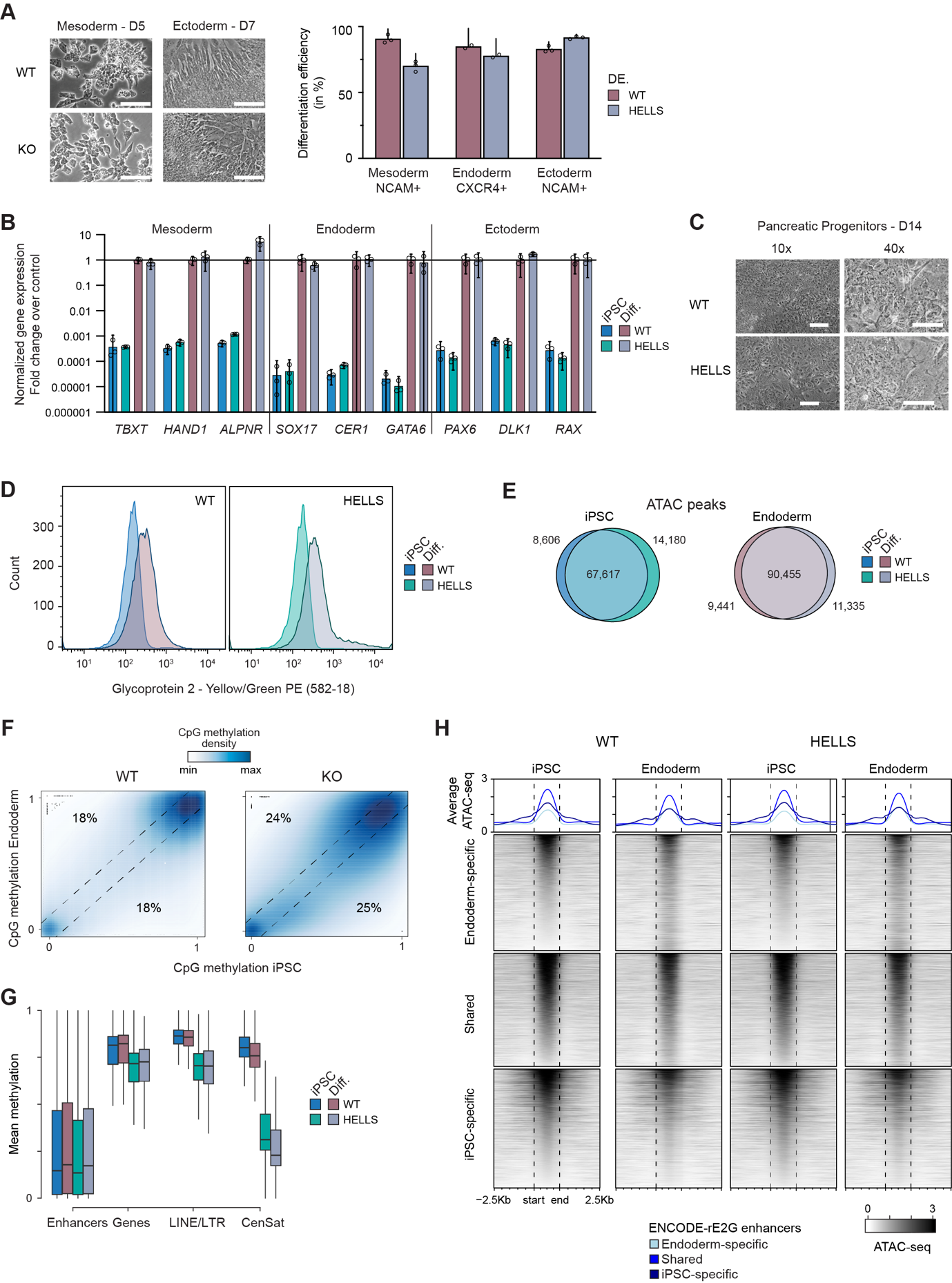


**Supplementary Figure 6. HELLS is not required to remodel enhancer landscapes essential for early embryonic lineage formation**

**A)** Directed differentiation of WT and HELLS KO iPSCs into mesoderm and ectoderm imaged by brightfield at 40x. The white scale bar reflects 25 µm (left panel). FACS-determined percentage of cells expressing the lineage-specific marker gene for each of the three germlayers (right panel). Representative brightfield images from one of the three independent replicates. From left to right: n (sum across all replicates) = 49,999; 55,675; 34,657; 33,636; 18,595; 20,244.

**B)** qPCR for three marker genes of each of the three germ layers (SOX17, CER1, and GATA6 for endoderm; TBXT, HAND1, and ALPNR for mesoderm; PAX6, DLK1, and LHX1 for ectoderm) in undifferentiated (light hue) and differentiated (dark hue) for WT (blue) and HELLS KO (green) cells. Data are normalized to the internal control gene GAPDH. Error bars represent the 95% confidence interval. qPCR was performed on three technical replicates for each of the three independent replicates. Each plotted point represents the mean of the three technical replicates for one independent replicate.

**C)** Directed differentiation of WT and HELLS KO iPSCs into pancreatic progenitor cells imaged in brightfield at 10x and 40x. The white scale bar reflects 25 µm.

**D)** FACS-plot of undifferentiated and directly differentiated pancreatic progenitor cells for WT (left panel, blue) and HELLS knockout cells (right panel, green) stained with a PE-conjugated anti-Glycoprotein2 antibody.

**E)** Venn diagrams showing the overlap in ATAC-seq peaks between WT and HELLS KO iPSCs (left panel) and endoderm (right panel).

**F)** Smooth scatter plots comparing individual CpGs of iPSC and endoderm for WT (left panel) and HELLS KO (right panel). The x-axis represents the methylation level of CpGs in HELLS arrested cells, while the x-axis represents the CpG methylation level either WT (left panel) or HELLS KO (right panel) iPSCs, while the y-axis represents the respective Endoderm CpG methylation levels. The color scale indicates the density of CpGs at a particular methylation level, with blue representing higher density. The dotted lines represent a threshold of 0.1 change in methylation levels between iPSC and endoderm.

**G)** Boxplots showing mean methylation over genomic features for WT and HELLS KO iPSCs and endoderm. The horizontal bar shows the median per feature, and boxes and whiskers reflect the quartiles. From left to right: n represents the number of regions included for calculating the DNA methylation distribution for each feature, with values of 16,201; 23,263; 1,411,943; 2,184.

**H**) Heatmap of ATAC-seq signal across ENCODE-rE2G iPSC-specific, Endoderm-specific, and shared enhancers for WT and HELLS KO iPSCs and endoderm.


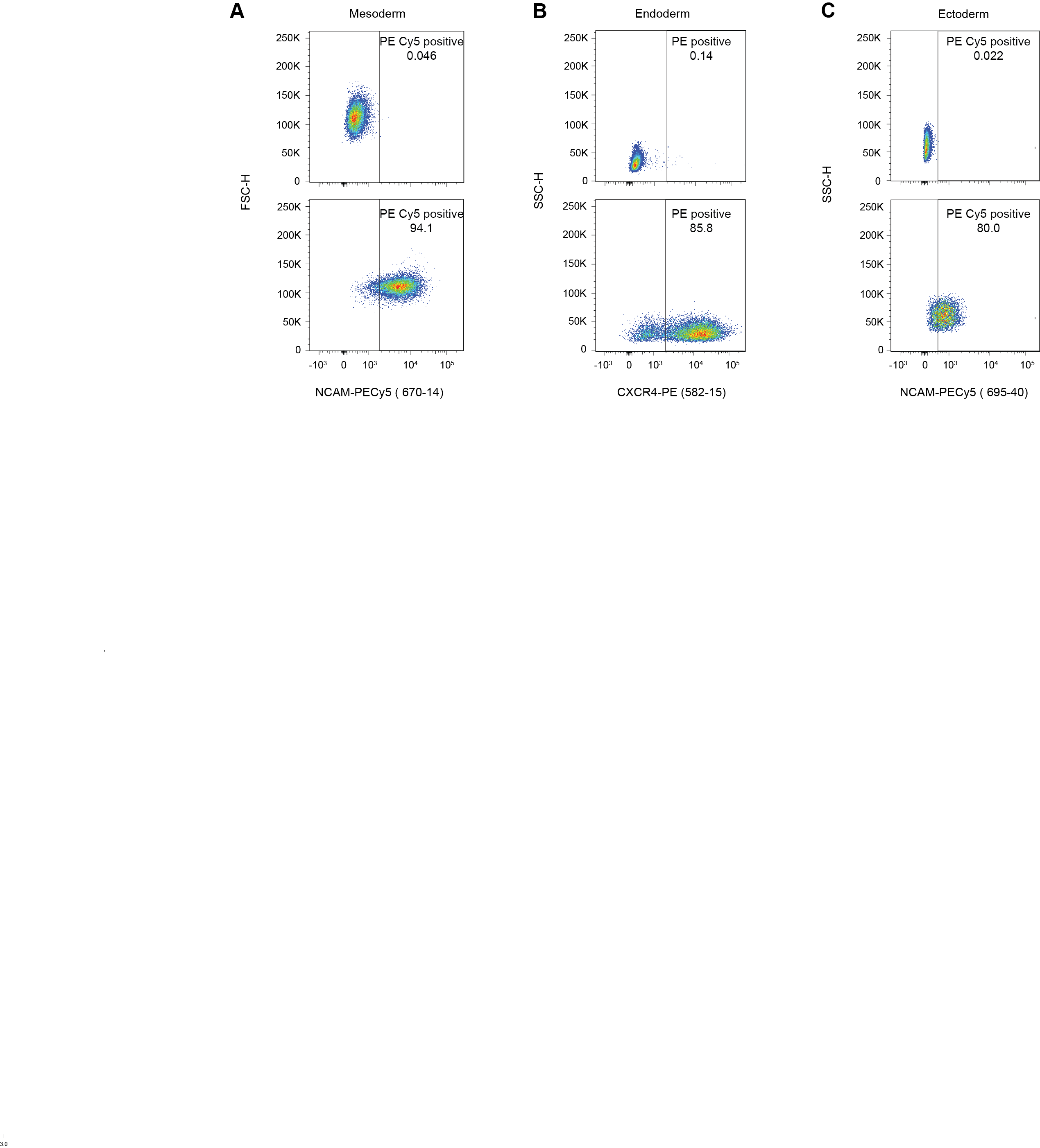


**Supplementary Figure 7: Gating strategy for sorting three-germ layer differentiated human iPSCs**

Percentage of sorted cells were analyzed using FlowJo. Live cells were selected using FSC-A/SSC-A, doublets were similarly removed with FSC-H/FSC-W and SSC-H/SSC-W. Cells were stained for the cell-type specific marker and undifferentiated control hiPSCs were analyzed to set the differentiation positive gate (top panel). Finally, the differentiated positive cells were sorted (bottom panel. **A**) Mesoderm, **B**) Endoderm, and **C**) Ectoderm.
