# Supplementary material for: HELLS is required for maintaining proper DNA modification at human satellite repeats
Source: Genome Biol. 2025 Jul 17;26:211. doi: 10.1186/s13059-025-03681-9 (PMC12273238; doi:10.1186/s13059-025-03681-9)
Supplement: Supplementary file 3 — Additional file 3. [file 13059_2025_3681_MOESM3_ESM.pdf]

Unprocessed image matching Figure 1A and Supplementary Figure 1D western blots

Always from left to right: WT* (p38), HELLS KO B3* (p8), HELLS KO H7(p8), WT (p38), WT (p30), DNMT3A KO (p10), DNMT3B KO (p10), DNMT3AB DKO (p10)

A

60 seconds

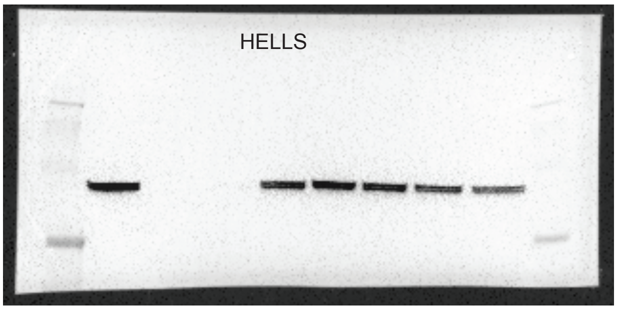

3 seconds

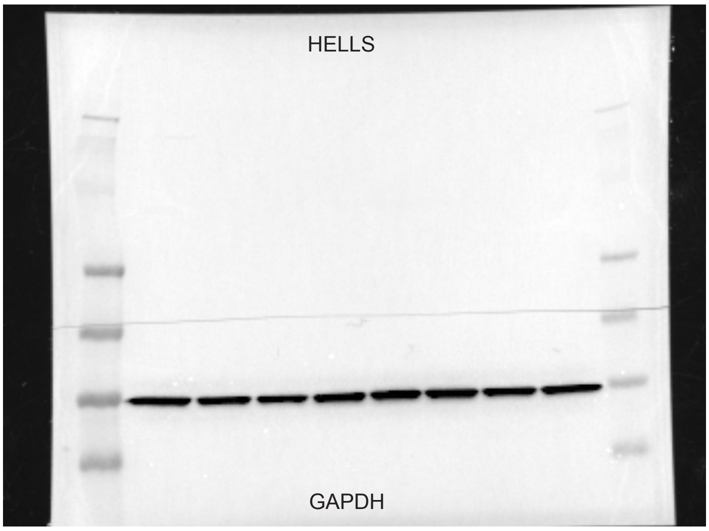

B

33.8 seconds

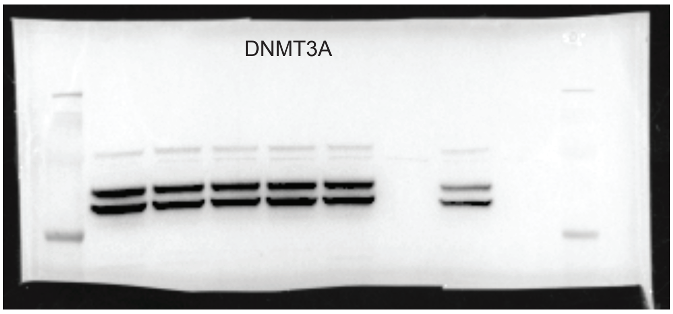

3.1 seconds

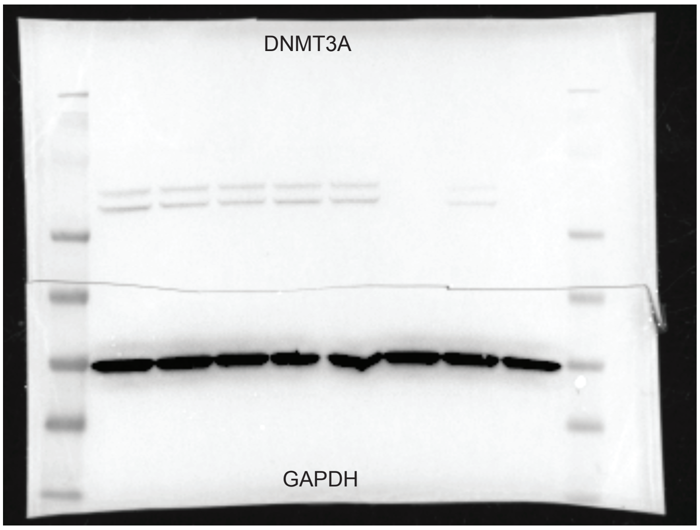

C

21.5 seconds

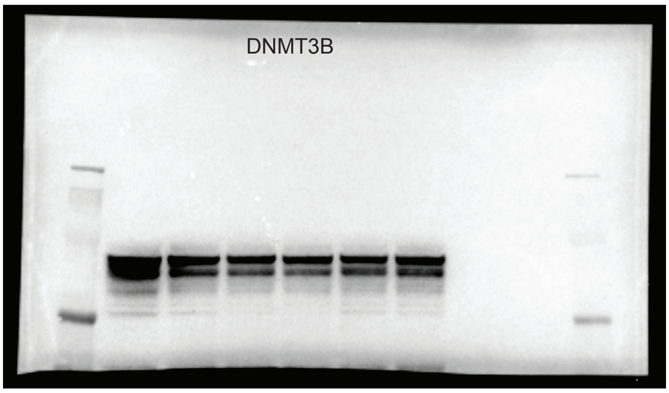

3.6 seconds

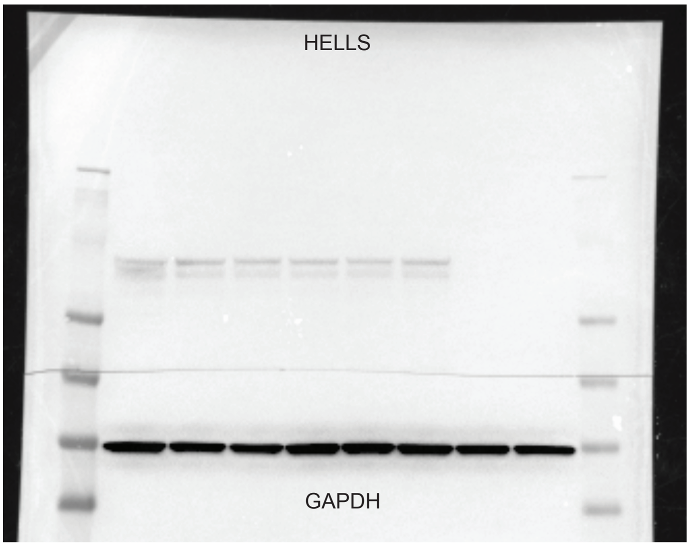

Guckelberger, Source data Figure 1A and Supplementary Figure 1D
