# Supplementary figures and images for: HELLS is required for maintaining proper DNA modification at human satellite repeats
Source: Genome Biol. 2025 Jul 17;26:211. doi: 10.1186/s13059-025-03681-9 (PMC12273238; doi:10.1186/s13059-025-03681-9)

A) First round of targeting

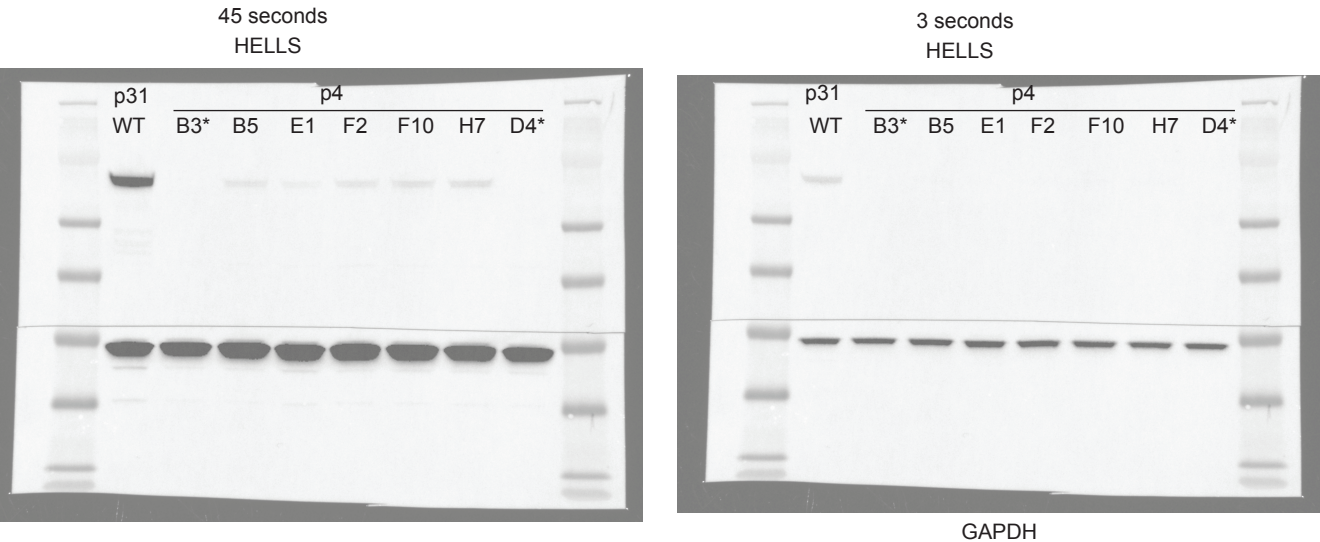

B) Second round of targeting

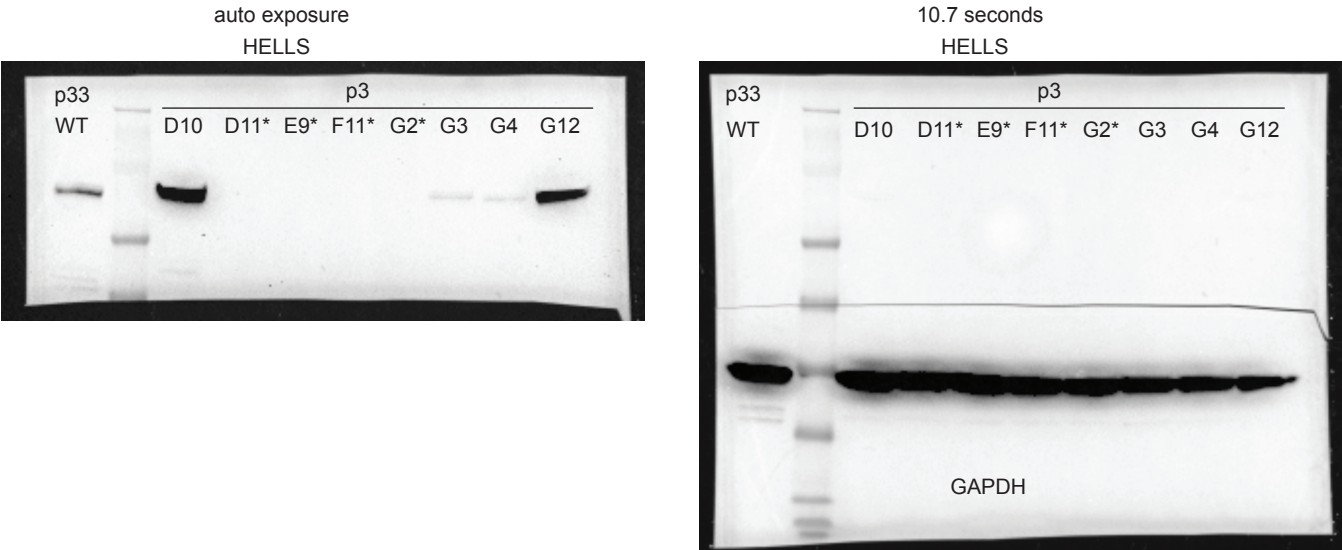

C) Third round of targeting

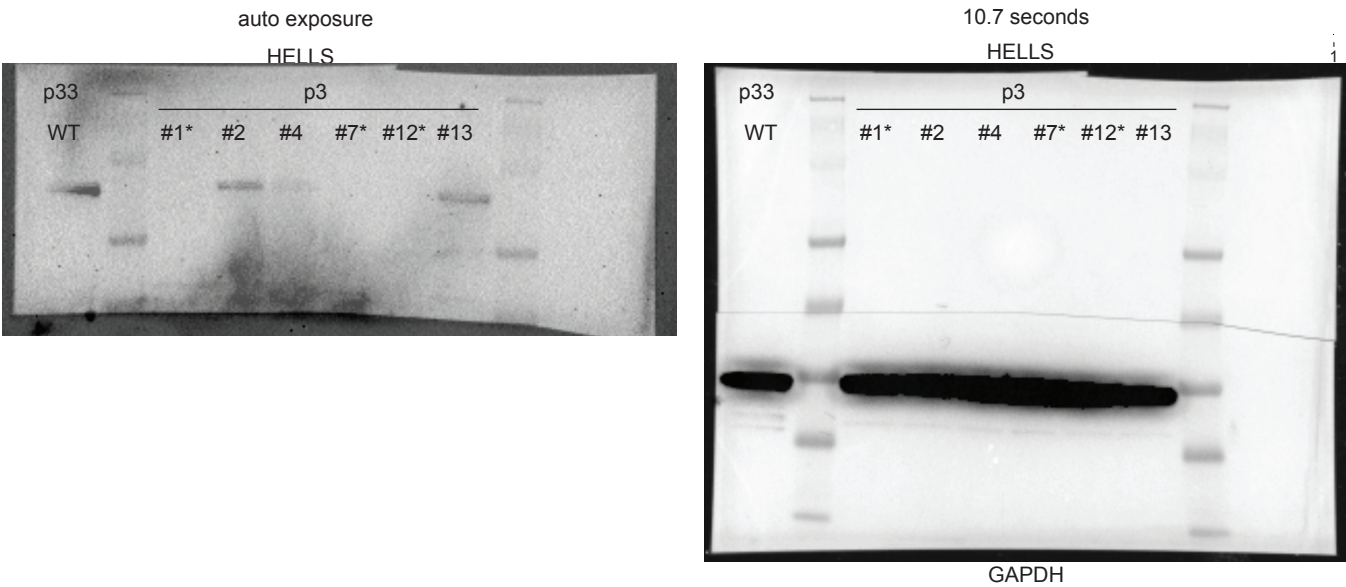

Supplement: Supplementary file 4 — Additional file 4. [file 13059_2025_3681_MOESM4_ESM.pdf]
